# Therapeutic and Improving Function of Lactobacilli in the Prevention and Treatment of Cardiovascular-Related Diseases: A Novel Perspective From Gut Microbiota

**DOI:** 10.3389/fnut.2021.693412

**Published:** 2021-06-07

**Authors:** Xin Zhao, Xinqin Zhong, Xiao Liu, Xiaoying Wang, Xiumei Gao

**Affiliations:** ^1^Ministry of Education Key Laboratory of Pharmacology of Traditional Chinese Medical Formulae, Tianjin University of Traditional Chinese Medicine, Tianjin, China; ^2^School of Chinese Materia Medica, Tianjin University of Traditional Chinese Medicine, Tianjin, China

**Keywords:** cardiovascular-related diseases, lactobacilli, gut microbiota, antimicrobials, therapeutic use

## Abstract

The occurrence and development of cardiovascular-related diseases are associated with structural and functional changes in gut microbiota (GM). The accumulation of beneficial gut commensals contributes to the improvement of cardiovascular-related diseases. The cardiovascular-related diseases that can be relieved by *Lactobacillus* supplementation, including hypercholesterolemia, atherosclerosis, myocardial infarction, heart failure, type 2 diabetes mellitus, and obesity, have expanded. As probiotics, lactobacilli occupy a substantial part of the GM and play important functional roles through various GM-derived metabolites. Lactobacilli ultimately have a beneficial impact on lipid metabolism, inflammatory factors, and oxidative stress to relieve the symptoms of cardiovascular-related diseases. However, the axis and cellular process of gut commensal *Lactobacillus* in improving cardiovascular-related diseases have not been fully elucidated. Additionally, *Lactobacillus* strains produce diverse antimicrobial peptides, which help maintain intestinal homeostasis and ameliorate cardiovascular-related diseases. These strains are a field that needs to be further investigated immediately. Thus, this review demonstrated the mechanisms and summarized the evidence of the benefit of *Lactobacillus* strain supplementation from animal studies and human clinical trials. We also highlighted a broad range of lactobacilli candidates with therapeutic capability by mining their metabolites. Our study provides instruction in the development of lactobacilli as a functional food to improve cardiovascular-related diseases.

## Introduction

Cardiovascular diseases and related diseases, such as hypercholesterolemia, hypertension, atherosclerosis, obesity, and diabetes, are the leading causes of death worldwide and continue to be an economic and health burden (1–3). Alterations in the composition and function of the gut microbiota (GM), known as dysbiosis, are linked to the occurrence and development of cardiovascular-related diseases. Tang et al. (4) summarized the GM composition of patients suffering from atherosclerosis, hypertension, obesity, and type 2 diabetes mellitus (T2DM) with high Firmicutes/Bacteroides ratio, trimethylamine-N-oxide (TMAO), short-chain fatty acids (SCFAs), and bile acids (BAs), as well as lipopolysaccharide (LPS) alterations. Recent research has demonstrated that the function of GM dysbiosis on cardiovascular-related diseases includes the following four aspects: inflammatory response due to the release of intestinal bacterial endotoxin, lipid metabolism abnormality, *in vivo* oxidative stress reaction, and tryptophan metabolism abnormality (5, 6).

Probiotic supplementation could lead to a reduction in cardiovascular risk (4, 7). Probiotics may act through different mechanisms, including establishing intestinal balance, affecting nutrient absorption and immune functions, and competing with pathogens (8). Among gut probiotics, the genus *Lactobacillus* is a Gram-positive probiotic classified as lactic acid bacteria (9). Various *Lactobacillus* strains have been widely studied for their interventions in cardiovascular-related diseases via the modulation of lipid cholesterol metabolism, immune-inflammatory response, oxidative stress response, and the involvement of GM-derived metabolites, including TMAO, SCFAs, LPS, and BAs (10–13). The metabolites produced by *Lactobacillus*, especially antimicrobial substances, can inhibit the growth of pathogens and regulate GM disorder (14, 15). They act as the protectors of GM and are the major contribution of lactobacilli in the improvement of cardiovascular-related diseases. However, as an important therapy for the improvement of cardiovascular-related diseases, a wide range of *Lactobacillus* can also be mined and visualized with a clear function. The discovery of antimicrobials by genome mining is feasible and worthwhile because the subsequent identification and isolation of known and putative molecules can attract pharmacological interest (16). The specific alteration of *Lactobacillus* through these mediators in the host physiology for the remedy of cardiovascular-related diseases should also be analyzed for future studies. Developing more *Lactobacillus* strains is beneficial for clinical application.

China issued the list of bacteria, including 13 *Lactobacillus* species, which are recognized as safe ingredients and widely used in the production of food products. The list was supplemented in the form of an announcement by the National Health Commission of the People's Republic of China [http://www.nhc.gov.cn, (2010) No. 65]. From 2005 to 2021, more than 20 strains among nine *Lactobacillus* species have been classified as “generally recognized as safe” by the U.S. Food and Drug Administration (https://www.accessdata.fda.gov). Besides, the European Food Safety Authority provides the assessment for probiotics, and 37 *Lactobacillus* species had been recommended for the Qualified Presumption of Safety list until 2021 (17–20). Among these species, *L. acidophilus, L. casei, L. crispatus, L. curvatus, L. delbrueckii, L. fermentum, L. gasseri, L. helveticus, L. johnsonii, L. paracasei, L. plantarum, L. reuteri, L. rhamnosus*, and *L. salivarius* were certified by at least two organizations (Supplementary Table 1). *L. murinus* has made a remarkable contribution to the prevention and treatment of hypertension (21). This review studied 38 *Lactobacillus* species (Supplementary Figure 1), updated the findings of the relationship between *Lactobacillus* and cardiovascular-related diseases, and provided a rich candidate of *Lactobacillus* strains that lessen cardiovascular risks.

## How do Lactobacilli as GM Commensal Alleviate Cardiovascular-Related Diseases?

**Figure 1:**
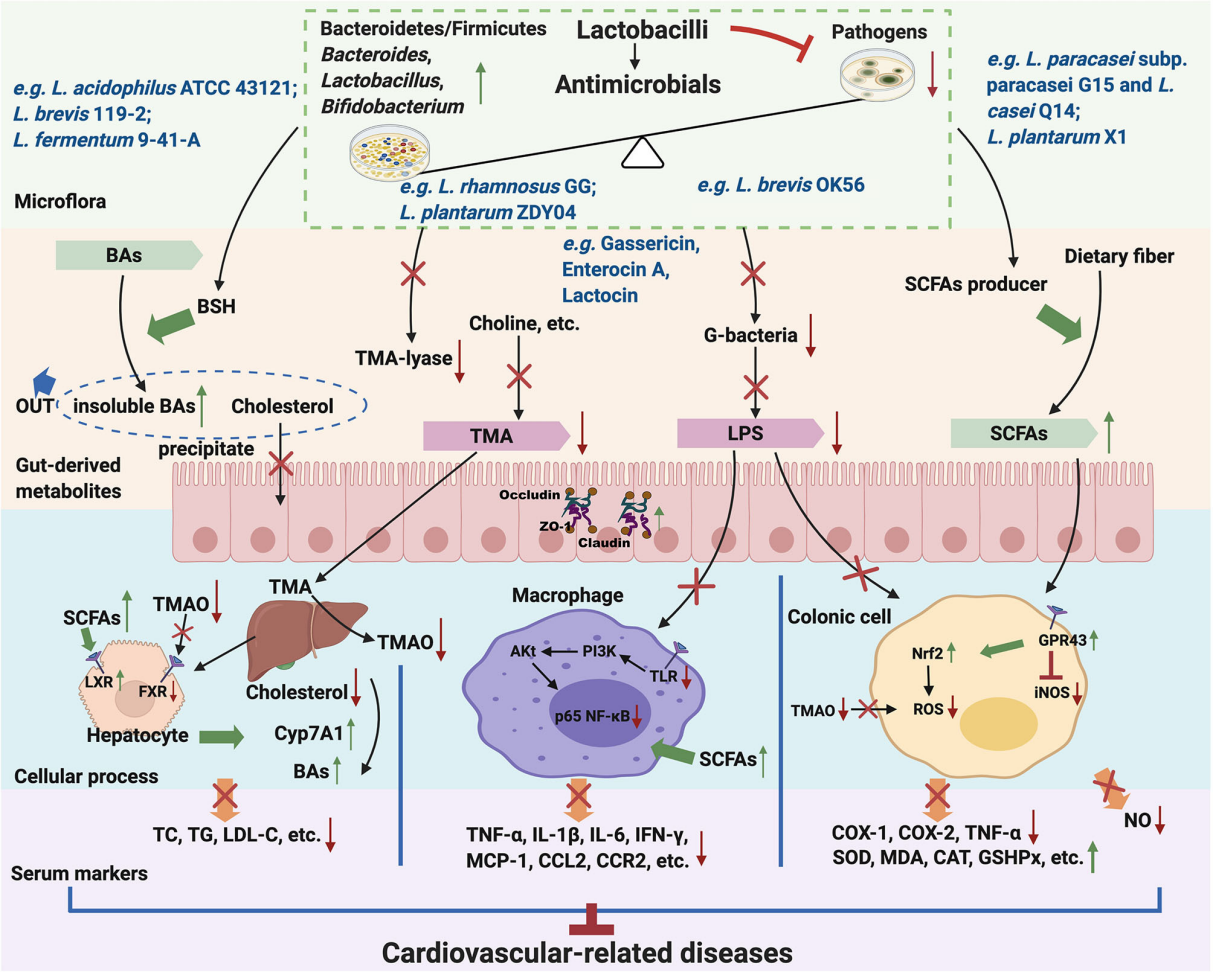
Mechanisms of lactobacilli on prevention and treatment of cardiovascular-related diseases through the GM. Green↑, Increase/Promote; Red↓/×/⊥, Decrease/Inhibit.

In recent years, our perception of the microbiome has evolved from a group of inert microorganisms into a true “endocrine organ” (22). The GM-dependent mechanism of *Lactobacillus* has also attracted widespread attention. A few reports revealed the evidence of lactobacilli on modulating GM composition, including only *L. acidophilus, L. brevis, L. casei, L. fermentum, L. johnsonii, L. mucosae, L. paracasei, L. plantarum, L. reuteri, L. rhamnosus, L. sakei, L. salivarius*. Nonetheless, the primary beneficial effect of lactobacilli starts from restoring GM abundance and species diversity. *Lactobacillus* colonization in the intestinal tract directly affects intestinal homeostasis and reduces gut permeability by inhibiting pathogens because of their antimicrobial products. *Lactobacillus* can modulate gut-derived metabolites and further decrease the level of serum cholesterol and reduce inflammation and oxidant damage. Many studies have revealed the relevant relationships between gut-derived mechanisms and the development of cardiovascular-related diseases (Figure 1).

## *Lactobacillus* Corrects Risk-Associated Microflora and Metabolites

Characteristic shifts in GM structure due to *Lactobacillus* intervention are manifested by corrections of vital gut dysbiosis parameters, such as decreased Firmicutes/Bacteroidetes ratio and abundant *Bacteroides, Lactobacillus*, and *Bifidobacterium* (Table 1). *Lactobacillus* further affects itself and other intestinal microbes that metabolize the host digestive products into various metabolites, such as trimethylamine (TMA), SCFAs, LPS, and BAs, which are involved in the progression of cardiovascular-related diseases (48). *Lactobacillus* reduces the putative cardiovascular risk mediator TMAO, which is produced by TMA oxidation in the liver, to prevent atherosclerosis and hypertension (11, 49). However, the possible mode of action of lactobacilli on TMAO is still unclear because a paucity of literature is available on the subject, and supporting clinical data is limited. Genomic data of GM showed that 37 bacterial species belonging to the phyla Firmicutes, Proteobacteria, and Actinobacteria harbor genes involved in TMA production (50). In consideration of the role of GM in TMAO metabolism, lactobacilli might inhibit gut microbes that produce key enzymes that catalyze TMA production. *Lactobacillus* supplementation promotes the SCFAs-producing bacteria *Roseburia, Ruminococcus*, and *Eubacterium* to facilitate the dietary fiber-fermented byproducts SCFAs (51, 52), which play critical roles in maintaining healthy cardiovascular functions. As the major component of the outer membrane of Gram-negative bacteria, LPS plays a key part in the pathogenesis of hypertension, obesity, and T2DM. *Lactobacillus* has the ability to reduce the LPS concentration in serum (53, 54). The potential of lactobacilli to inhibit Gram-negative is discussed in this paper. Disorders in BAs metabolism cause dyslipidemia, cardiovascular diseases, and diabetes. Lactobacilli have a major function in BAs biotransformation by promoting the activity of microbial bile salt hydrolase (BSH), regenerating primary free BAs, and facilitating the microbial formation of secondary BAs, as well as a range of intermediates (55, 56).

**Table 1:** A variety of lactobacilli with therapeutic effects on cardiovascular-related diseases.

***Lactobacillus***	**Strain**	**Via Cellular process**			**Via Gut Microbiota (GM)**		**Cardiovascular-related diseases**	**Model**	**Reference**
		**Cholesterol-lowering**	**Anti-Inflammation**	**Anti-oxidative stress**	**Metabolite**	**GM variety**			
*L. acidophilus*	La5	•	°	°	°		Dyslipidemia	Type 2 diabetic adults	(23)
*L. amylovorus*	CP1563	•	°	°	°	*Roseburia* and *Lachnospiraceae*↑ *Collinsella*↓	Obesity	Obese class I adults; pre-obese healthy adults	(24, 25)
*L. casei*	01	°	°	°	°		Type 2 diabetes mellitus	Type 2 diabetic adults	(26)
	Shirota	•	°	°	SCFAs↑	*Bifidobacterium, Bacteroides fragilis* group, *Atopobium cluster, Lactobacillus gasseri* group↑	Obesity	Obese children	(27)
*L. fermentum*	ME-3	•	•	•	°		Cardiovascular and diabetes risk	Asymptomatic adults	(13)
*L. gasseri*	BNR17	°	°	°	°		Obesity	Overweight and obese adults	(28)
	SBT2055(LG 2055)	°	°	°	°		Obesity	Healthy adults with large visceral fat areas	(29, 30)
		•	°	°	°		Obesity and type 2 diabetes mellitus	Hypertriacylglycerolemic adults	(31)
*L. helveticus*	LBK-16H	°	°	°	°		Hypertension	Hypertensive adults	(32, 33)
	CM4	°	°	°	°		Hypertension	Hypertensive adults	(34)
*L. murinus*		°	°	°	°		Salt-sensitive hypertension	*In vitro* trial (TH17 cells) healthy men, HSD-fed FVB/N mice	(21)
*L. plantarum*	Lp299v	°	•	•	°		Coronary artery disease	Men with stable coronary artery disease	(35)
	ECGC13110402	•	•	°	°		Hypercholesterolemia	Hypercholesterolemic adults	(36)
	OLL2712	•	•	°	°		Obesity	Overweight adults	(37)
	Dad-13	°	°	°	°	Bifidobacteria and Lactobacilli↑ Enterobacteriaceae and *Staphylococcus*↓	Obesity	Overweight adults	(38)
*L. reuteri*	NCIMB30242	•	•	°	°		Hypercholesterolemia	Hypercholesterolemic adults	(7)
	DSM17938	•	°	°	BAs↑	Diversity↑	Insulin sensitivity	Type 2 diabetic adults	(39)
	ADR-1 or ADR-3	•	°	°	°	*Lactobacillus* and *Bifdobacterium*↑	Type 2 diabetes mellitus	Type 2 diabetic adults	(40)
	V3401	°	•	°	°	Verrucomicrobia↑	Metabolic syndrome	Adults with metabolic syndrome	(41)
*L. rhamnosus*		°	°	°	°		Myocardial infarction	Post-myocardial infarction adults	(42)
	GG	°	°	°	°		Type 2 diabetes mellitus	Middle age and older adults	(43)
	GG	°	•	°	LPS↓		Coronary artery diseases	Adults with coronary artery diseases	(44)
		°	•	•	°		Myocardial infarction	Post-myocardial infarction adults	(45)
*L. sakei*	CJLS03	°	°	°	°		Obesity	Obese adults	(46)
*L. salivarius*	Ls-33	°	°	°	°	*Bacteroides-Prevotella-Porphyromonas* group/Firmicutes↑	Obesity	Obese adolescents	(47)

*°, Non-significant effect between groups/Not applicable; •, Observed effect; ↑, Increase/Promote; ↓, Decrease/Inhibit*.

## *Lactobacillus* Inhibits Pathogenic Bacteria and Reduces Gut Permeability

The destruction of the intestinal barrier function in patients with cardiovascular-related diseases is the main reason for the excessive proliferation of related pathogens and the increase in plasma endotoxin concentration. These conditions further aggravate gut permeability, promote inflammation, and increase cardiovascular risks (57). *Lactobacillus* exerts strong antimicrobial activity against pathogens and reinforces the intestinal barrier (58). Although antimicrobial production by lactobacilli has been regarded as a beneficial trait for some time, the full extent of the benefits of antimicrobials in the gut is only beginning to be appreciated. A comparison of *L. salivarius* UCC118 (with bacteriocin Abp118) with the non-bacteriocin-producing strain, *L. salivarius* UCC118 (knock out Abp118), showed that antimicrobial production resulted in an increase in Bacteroidetes and a reduction in the proportions of Actinobacteria in the GM of diet-induced obesity mice (59). Further clinical research demonstrated that the favorable effects of the UCC118 strain possibly rely on positive alterations in gut permeability and microbiota (60). An animal study showed that *L. plantarum* ZLP001 pretreatment alleviated the reduction in junction proteins (claudin-1, occludin, and ZO-1) (61), which are the key contributors to establishing an effective intestinal barrier (62). Several *in vitro* experiments also showed that lactobacilli positively affect gut permeability, as incubation of Caco-2 cells with different *Lactobacillus* strains was found to restore impaired intestinal barrier (63, 64).

Of note, LPS produced by Gram-negative bacteria in the gut is the main component of endotoxin. Gassericin, enterocin A, paracin 1.7, lactocin, bacteriocin TSU4, and bacteriocin 217 greatly inhibit Gram-negative bacteria (such as *Escherichia coli*) (65–74). Therapeutic manipulation of microbiota using different antimicrobial strategies may be a useful approach for the management of cardiovascular-related diseases. Our extended list of antimicrobial compounds demonstrates that lactobacilli are excellent antimicrobial producers (Supplementary Table 2). *L. paracasei, L. sakei, L. plantarum, L. casei, L. gasseri, L. alimentarius, L. coryniformis, L. panis, L. crispatus, L. johnsonii, L. amylovorus, L. curvatus, L. rhamnosus*, and *L. helveticus* can now readily be tapped experimentally for improving cardiovascular-related diseases.

## *Lactobacillus* Presents Cholesterol-Lowering, Anti-Inflammation, and Anti-Oxidative Stress Effects

By expressing BSH, lactobacilli have certain advantages in the intestine that result in the deconjugation of free bile salts, which combine with cholesterol to form a precipitate and are more easily excreted via feces (10, 75–77). Lactobacilli induce the catabolism of cholesterol to BAs by cytochrome P450 a1 (Cyp7A1) by inhibiting farnesoid X receptor (FXR) (78, 79) and inhibit the reabsorption of BAs into the enterohepatic circulation (80). SCFAs are transported to the liver to modulate the hepatic metabolism of lipids and cholesterol by elevating the transcriptional activity of liver X receptor (LXR) alpha and upregulating Cyp7A1 (81). By contrast, TMAO regulates changes in BAs synthesis to accelerate aortic lesion formation by activating FXR and small heterodimer partner to suppress BAs synthetic enzyme expression and decrease BAs transporters in the liver but not in the gut (82).

*Lactobacillus* has anti-inflammatory activity and exerts protective effects on damage by inhibiting PI3K/Akt, NF-κB activation, and inflammatory cytokines, such as tumor necrosis factor-α (TNF-α), interleukin (IL)-1β, IL-6, IL-8, and monocyte chemotactic protein 1 (MCP-1). *Lactobacillus* promotes SCFAs production that activates the G protein-coupled receptor 43 (GPR43) pathway (51, 83), and downregulates the expression and activation of NF-κB, interferon-γ, Toll-like receptor 2, TNF-α, and other cytokines/chemokines involved in inflammatory responses (84). *Lactobacillus* presents an anti-oxidative effect by blocking LPS-induced nitric oxide (NO) production; decreasing the expression of cyclooxygenase (COX)-1, COX-2, inducible nitric oxide synthase (iNOS), and TNF-α mRNA (85). In addition, *Lactobacillus* promotes Nrf-2-induced antioxidative activity in mice to reduce cardiovascular risk (86). Other serum markers of oxidative stress, including thiobarbituric acid-reactive substances (TBARS), superoxide dismutase (SOD), malondialdehyde, catalase (CAT), glutathione peroxidase (GSH-Px), norepinephrine (NE), and prostacyclin, were also altered by *Lactobacillus* intervention. Butyrate promotes endothelial nitric oxide synthase (eNOS) expression and NO bioavailability in vascular smooth muscle cells (87). TMAO induces reactive oxygen species (ROS) generation, particularly mitochondrial ROS through the suppression of SOD2 activity and sirtuin-3 in human umbilical vein endothelial cells and aortas from apolipoprotein E (ApoE)-deficient mice.

Without a doubt, our review is limited by the available studies. A few metabolites such as indole derivatives, polyamines, and taurine are not deeply discussed in the function of lactobacilli treatment. Furthermore, some new regulatory mechanisms of vascular-related diseases should be concerned, such as activation of aromatic hydrocarbon receptor, rather than classical pathways.

## *Lactobacillus* Strains Worthy of Attention and Their Therapeutic Use

Various lactobacilli strains play roles in reducing cardiovascular risk factors, balancing metabolic disorders, and altering health-related microflora metabolite production. However, little is known about the role of these supplements as important dietary components in preventing or treating cardiovascular-related disease. Still, some reports and clinical studies were conducted, offering new ways of treatment. In addition, some lactobacilli species, such as *L. acidophilus, L. gasseri*, and *L. rhamnosus*, have been associated with a wide range of purported health benefits, such as anti-infectious activity; immunomodulation; anti-allergenic effects; and tumor suppression. Below are the selected lactobacilli that improve cardiovascular-related diseases. Eleven *Lactobacillus* species take effect on extensive cardiovascular risks, 10 species act on a single disease, and the effects of the other 17 species on cardiovascular-related diseases have not been reported (Supplementary Figure 1). Due to the strain specificity and individual physical differences, a personalized clinical evaluation and intake recommendation should be developed.

### Lactobacillus acidophilus

*L. acidophilus*, which is one of the most important resident microorganisms in the small intestine, has a cholesterol-lowering function for the improvement of hyperlipidemia, hypercholesterolemia, atherosclerosis, coronary heart disease (CHD), T2DM, and obesity (75, 88–92). Strain ATCC 43121 can reduce cholesterol metabolism by increasing insoluble BAs (lithocholic acid) in hypercholesterolemia rats (75). *L. acidophilus* ATCC 4356 reduced the expression of the Niemann-Pick C1-Like 1 and glucose transporter 2 gene and inhibited the cellular uptake of micelle cholesterol and glucose in Caco-2 cells (89, 93). Further animal studies (90, 94) found that the administration of strain ATCC 4356 can prevent atherosclerosis by inhibiting the absorption of intestinal cholesterol and enhancing the abundance of *Lactobacillus* and *Bifidobacterium* in the gut. In addition to reducing total cholesterol (TC), *L. acidophilus* SJLH001 isolated from fermented food can also reduce blood glucose in high-fat diet (HFD)-induced obese mice by regulating the key genes involved in the glucose transport, ion channels, and immune response of the bacterium (95) and affect the structure of intestinal microbiota. Strain LA5 improved saturated fat-induced obesity mouse model through the enhanced intestinal *Akkermansia muciniphila* (96). Besides, *L. acidophilus* KLDS1.0901 administration showed antidiabetic and antioxidant activity in T2DM mice induced by HFD and intraperitoneal injection of streptozotocin (STZ) (97). *L. acidophilus* is known for the mixed-use with Bifidobacteria in probiotic dairy foods and effectively used in clinical practice (23). Since *L. acidophilus* is safe in humans, it is likely a potential drug for improving cardiovascular health. Therefore, clinical studies are warranted to explore the beneficial effects of this bacterium.

### Lactobacillus brevis

*L. brevis* is a microaerophilic, obligately heterofermentative lactic acid bacterium isolated from various natural environments with relieving effects on hypercholesterolemia, atherosclerosis, obesity, and hypertension. The potential mechanism of the cholesterol-lowering effect of *L. brevis* 119-2 was the inhibition of 3-hydroxy-3-methylglutaryl-CoA reductase activity by insulin-induced gene (Insig) protein and the induction of the catabolism of cholesterol to BAs by Cyp7A1 (78). *L. brevis* OK56 ameliorated HFD-induced obesity in mice by inhibiting NF-κB activation and gut microbial LPS production (98). *L. brevis* OPK-3 inhibited the induction of inflammation in adipose tissue along with preventing weight gain (99). *L. brevis* presented an anti-hypertensive effect by blocking LPS-induced NO production and decreasing the expression of COX-1, COX-2, iNOS, and TNF-α mRNA (85). Gamma-aminobutyric acid-producing strains, *L. brevis* DPC6108, and *L. brevis* DSM32386, had the potential to improve metabolic profiles in mice with metabolic dysfunction (100). There are few studies on the clinical practicability and suggestions for *L. brevis*, and extensive studies are expected.

### Lactobacillus casei

*L. casei* is a transient bacterium in the human body and can relieve hypocholesterolemia, atherosclerosis, and hypertension in mice/rats through a cholesterol-lowering mechanism attributed to the improvement of BAs, SCFAs, and TMAO (10, 101–103). *L. casei* 01 supplementation significantly decreased dietary intake and body weight through improving serum fetuin-A and sirtuin1 levels and glycemic response in T2DM patients (26). *L. casei* can also effectively treat T2DM and hyperglycemia by suppressing GM-mediated inflammation (104–106). *L. casei* prevented T2DM possibly via a microbiota-based BA-chloride exchange mechanism by upregulating chloride ion-dependent genes (*ClC1-7, GlyR*α*1, SLC26A3, SLC26A6, GABAA*α*1, Bestrophin-3*, and *CFTR*) (104). *L. casei* CCFM419 had a potential ability to ameliorate insulin resistance and hyperglycemia in T2DM mice through underlying PI3K/Akt signaling pathway and gut flora-SCFAs-inflammation/GLP-1 mechanism (105, 106). *L. casei* strain Shirota as a dietary intervention not only played a role in controlling childhood obesity and improving lipid metabolism through an apparent increase in acetic acid concentration (27). The common points of these lactobacilli in modulating GM were the increase of *Lactobacillus* and *Bifidobacterium*. *L. casei* C1 supplementation in hypertensive rats increased serum glutathione (GSH) and NO levels; thus, the strain worked through antioxidant function and increased NO levels (induced vasodilation) for attenuating hypertension (107). Besides, *L. casei* has a significant effect on the treatment of liver injury (108), indicating that this bacterium is suitable for patients with the above risks.

### Lactobacillus delbrueckii

*L. delbrueckii* subsp. *bulgaricus* (*L. bulgaricus*) is widely used in the dairy industry and can treat hypercholesterolemia, ischemic heart disease, and diabetes. *L. bulgaricus* NS12 can reduce serum TC, low-density lipoprotein (LDL), apolipoprotein B, and free fatty acid levels and increase apolipoprotein A-I levels in rats with high cholesterol diet. Simultaneously, this strain remarkably reduced liver damage and liver lipid deposition by regulating the mRNA expression levels of liver enzymes related to cholesterol metabolism (109). Besides, *L. bulgaricus* had a certain protective effect on the heart. *L. bulgaricus* 51 remarkably reduced rapid arrhythmia after reperfusion in ischemic rats, reduced the release of NE and prostacyclin in the first minute of reperfusion, and improved the heart function of ischemic rats. The protective effect is related to the activation of CAT and the expression of heat shock protein 70 (110, 111). *L. delbrueckii* subsp. *lactis* PTCC1057 treatment decreased the fasting blood glucose and fetuin-A level and increased the serum sestrin 3 level in the STZ-induced diabetic mice (112). However, whether *L. delbrueckii* is an effective supplement or not in clinical cohorts is still being debated. Nonetheless, this bacterium has shown promising results in clinical trials for patients with respiratory or vaginal infections (113, 114).

### Lactobacillus fermentum

*L. fermentum* colonizes the gut and plays an important role in intestinal health. Clinical and animal experiments have proven its preventive and therapeutic effects on hypercholesterolemia, hyperlipidemia, hyperglycemia, atherosclerosis, obesity, and hypertension. *L. fermentum* has diverse regulatory mechanisms in addition to cholesterol-lowering metabolism together through SCFAs and BAs regulation (76, 115–120). Adverse physiological alterations, including considerably higher levels of serum TC, low-density lipoprotein cholesterol (LDL-C), triacylglycerols (TG), atherogenic index, coronary artery risk index, hepatic lipids, lipid peroxidation, mRNA expression of inflammatory cytokines (TNF-α and IL-6) in the liver, and anti-oxidative enzyme activities (CAT, SOD, and GSH-Px) in the liver and kidney, improved after the supplementation of *L. fermentum* MTCC: 5898-fermented buffalo milk (2.5% fat) in rats fed with cholesterol-enriched diet (118). *L. fermentum* can also play a role in fat metabolism. Fecal cholesterol and BAs levels considerably increased after *L. fermentum* 9-41-A administration (121). Intestinal *Lactobacillus* and *Bifidobacterium* colonies increased whereas *Escherichia coli* colonies decreased. *L. fermentum* strains can effectively inhibit HFD-induced obesity through modulation of the PPAR-α signaling pathway, oxidative phosphorylation in adipose tissue, and gut microbiome (122–124). *L. fermentum* CECT5716 can also restore vascular redox status and improve eNOS coupling to prevent hypertension and endothelial dysfunction caused by tacrolimus (125). In clinical trials, the use of *L. fermentum* ME-3 positively affected blood lipoprotein, oxidative stress, and inflammatory profile (13). However, *L. fermentum* may destroy the intestinal barrier, so further safety evaluation should be carried out (126).

### Lactobacillus gasseri

Obesity is one of the common cardiovascular-related diseases, and research on new probiotic therapy has good application prospects. As a type of *Lactobacillus* found in the gastrointestinal tract, *L. gasseri* presents an anti-obesity effect by inhibiting lipid absorption. The effect of *L. gasseri* SBT2055 (LG2055) on fat hydrolysis was measured by measuring the activity of pancreatic lipase and the *in vitro* properties of the fat emulsion. The results showed that LG2055 increased the size of fat emulsion droplets and therefore inhibited lipase-mediated fat hydrolysis and promoted human fecal fat excretion (127). Similarly, skimmed milk fermented by LG2055 remarkably reduced average adipocyte size and reduced leptin and cholesterol in rats (128). LG2055 also has an anti-inflammatory function. A 24-week study found that the supplemental feeding of strain LG2055 to mice fed with a 10% fat diet could reduce the expression of pro-inflammatory genes, such as *CCL2* and *CCR2*, and prevent weight gain and fat accumulation (129). Consumption of probiotic LG2055 can reduce serum non-esterified fatty acid levels after meals and on an empty stomach, indicating that it may help reduce the risk of obesity and T2DM (29–31, 130). LG2055 and *L. gasseri* BNR17 also have a good anti-obesity effect on overweight and obese adults, and healthy adults with large visceral fat areas (28, 30, 130, 131). The cholesterol-lowering effect of *L. gasseri* SBT0270 in hypercholesterolemia rats is attributed to the inhibition of BA reabsorption into the enterohepatic circulation and the enhancement of the excretion capacity of acidic steroids in the feces, which can effectively reduce heart vascular risk (80). These findings warrant a subsequent longer-term prospective clinical investigation with a larger obese population.

### Lactobacillus helveticus

*L. helveticus* commonly used for dairy fermentation has long-term hypotensive effects in patients with hypertension (32). Two tripeptides (Val-Pro-Pro and Ile-Pro-Pro) that inhibit the activity of angiotensin I converting enzyme are produced in fermented milk and do not cause adverse effects while lowering blood pressure (34). Long-term intervention can reduce arterial stiffness in patients with hypertension (33). *L. helveticus* KII 13 isolated from fermented milk can produce hypotensive peptides, reduce serum cholesterol and, increase the expression of LDL receptor and *SREBF2* genes related to cholesterol metabolism in the liver in mice in the HFD group (132). Moreover, *L. helveticus* can absorb a certain degree of cholesterol for biotransformation *in vitro* (133). Also, the effectiveness study of *L. helveticus* on patients with hypertension and major depressive disorder requires further study (134).

### Lactobacillus paracasei

*L. paracasei*, a gastrointestinal tract bacterium, plays multiple roles in the improvement of cardiovascular-related diseases, including hypercholesterolemia, hyperlipidemia, atherosclerosis, T2DM, obesity, and hypertension. The cholesterol-lowering effect of *L. paracasei* NTU101 resulted in the increased abundance of *Allobaculum* and *Clostridium* XIVa (135). Supplementation with *L. paracasei* NTU101 considerably reduced the ratio of LDL-C to high-density lipoprotein cholesterol (HDL-C), SOD activity, and total antioxidant status of the blood and relieve the degree of TBARS; hence, this strain can effectively prevent hyperlipidemia-induced oxidative stress and atherosclerosis (136). *L. paracasei* NTU101 can also act in hypertension treatment through substances, such as angiotensin-converting enzyme inhibitors (ACEI) and aminobutyric acid (γ-aminobutyric acid) (137), and exert neuroprotection in the brain (138). *L. paracasei* can regulate cholesterol metabolism, BAs homeostasis, as well as the LXR/inflammatory axis of LPS-stimulated alveolar macrophages, in animals fed with HFD (139–142). *L. paracasei* also protects the cardiovascular system by regulating blood glucose, insulin sensitivity, and fat metabolism (143). Researchers evaluated the α-glucosidase inhibitory activity of eight *L. paracasei* strains *in vitro*, and *L. paracasei* TD062, which has a high α-glucosidase inhibitory activity (31.9%), showed excellent antidiabetic ability. Further *in vivo* study showed that *L. paracasei* TD062 had a positive effect on the antioxidant capacity and the expression levels of genes that were related to glucose metabolism and the PI3K/Akt pathway in diabetic mice (144). Similarly, the studies are in animal models, and extrapolation in humans needs further studies, especially patients associated with allergic disease (145).

### Lactobacillus plantarum

*L. plantarum* is a widespread member of the genus *Lactobacillus* and is commonly found in fermented food products and anaerobic plant matter. *L. plantarum* has important effects in preventing hypercholesterolemia, hyperlipidemia, atherosclerosis, T2DM, obesity, and hypertension. *L. plantarum* can absorb cholesterol directly from the culture medium and was thus selected as a probiotic that potentially reduced cholesterol levels in mice/rats (86, 146–154). *L. plantarum* HT121 improved serum lipid profiles, restored beneficial gut microbes, and regulated BAs metabolism (155). *L. plantarum* DR7 reduced cholesterol via the phosphorylation of AMPK, which downregulated the mRNA expression of 3-hydroxy-3-methyl glutaryl coenzyme A reductase in hepatic (HepG2) and intestinal (HT-29) cells (146). *In vivo* experiments confirmed that *L. plantarum* CAI6 and SC4 can regulate lipid metabolism and Nrf-2-induced oxidative defense in hyperlipidemic mice to reduce cardiovascular risk (86). *L. plantarum* ZDY04 remarkably reduced serum TMAO levels and TMAO-induced atherosclerosis by modulating the relative abundance of the families Lachnospiraceae, Erysipelotrichaceae, and Bacteroidaceae and the genus *Mucispirillum* in mice (11). *L. plantarum* strains can also exert anti-obesity effect by ameliorating lipid accumulation, oxidative damage, inflammation, and gut dysbiosis (156–158). *In vitro*, the cell-free supernatant of *L. plantarum* X1 can inhibit α-glucosidase activity and show potential antidiabetic ability. *L. plantarum* X1 can partially enhance antioxidant capacity and improve the secretion of cytokines and pancreatic damage in T2DM mice. In addition, this strain remarkably restored the acetic acid level and increased the butyric acid level in the feces of diabetic mice; thus, the ability of *L. plantarum* to lower blood sugar was closely related to exercise and fatty acid and intestinal flora composition changes (159). *L. plantarum* can also regulate blood pressure by inhibiting ACEI activity and promoting NO production and therefore improved learning and memory in rats with hypertension-induced vascular dementia induced by deoxycorticosterone salt (160). The expression of TNF-α, IL-6, MCP-1, vascular cell adhesion molecule, intercellular adhesion molecule, and E-selectin were remarkably downregulated in *L. plantarum* Lp91-fed LPS-induced mice compared with the control group; hence, its anti-inflammation effect might be involved in cardiovascular-related diseases (161). Besides, beneficial effects of *L. plantarum*, inulin, or their combination on GM, cardiac apoptosis, and diabetes have been studied (162–164). In the clinic, strain Lp299v improved vascular endothelial function and decreased systemic inflammation, and ECGC 13110402 exerted lipids reduction function (35, 36). Heat-treated *L. plantarum* OLL2712 reduced abdominal fat accumulation and chronic inflammation in overweight adults (37). GM analysis indicated that *L. plantarum* Dad-13 intervention in obese adults decreased the Firmicutes population and increased the Bacteroidetes population (38). Circulating gut-derived metabolites TMAO and SCFAs likely contribute to these improvements by *L. plantarum* and merit further study.

### Lactobacillus reuteri

*L. reuteri* has been reported to exist naturally in the intestines of all vertebrates and mammals and is associated with most cardiovascular-related diseases. *L. reuteri* has cholesterol-lowering effects in the body and can effectively reduce TC, TG, and LDL values (165–169). Similar to *L. plantarum* DR7, *L. reuteri* NCIMB30242 exerted cholesterol-lowering properties along the AMPK pathway (146). In clinical trials, *L. reuteri* NCIMB30242 can best meet the dietary requirements for therapeutic lifestyle changes (TLC), which remarkably reduced LDL-C and TC and improved other CHD risk factors, such as inflammatory biomarkers (7). A high-calorie diet led to heart damage and promoted heart failure, whereas oral *L. reuteri* GMNL-263 treatment can regulate plasma lipids and reduce high-calorie-induced cardiac inflammation, hypertrophy, and fibrosis (170, 171). In addition, *L. reuteri* GMNL-263 can treat obesity in high-energy diet-induced obese rats by improving serum levels of pro-inflammatory factors and antioxidant enzymes and remodeling white adipose tissue (WAT) energy metabolism (172). *L. reuteri* strain ATCC PTA 4659 may partially prevent diet-induced obesity through a previously unknown mechanism that induced the expression of carnitine palmitoyltransferase 1a in the liver (173). *L. reuteri* GMN-32 treatment can reduce the impact of diabetes on the heart, decrease blood glucose levels, inhibit caspase 8-mediated apoptosis, promote heart function, and prevent diabetic cardiomyopathy (174). *L. reuteri* strains ADR-1 and ADR-3 ameliorated symptoms of T2DM patients, and increased intestinal level of *L. reuteri* to further up-regulate *Lactobacillus* and *Bifidobacterium*, and decrease Bacteroidetes (40). Oral *L. reuteri* DSM17938 supplementation for 12 weeks did not affect glycated hemoglobin but improved insulin sensitivity and increased serum secondary BA deoxycholic acid levels in patients with insulin-treated T2DM (39). Furthermore, *L. reuteri* V3401 can reduce inflammatory markers in patients with metabolic syndrome, including obesity, and improve microbial intestinal composition, especially Verrucomicrobia, for improving metabolic syndrome and reducing cardiovascular risk mechanism (41). *L. reuteri* has achieved remarkable results in the treatment of osteoporosis (175), suggesting that it might improve bone and cardiovascular health by modulating the GM.

### Lactobacillus rhamnosus

*L. rhamnosus* is one of the most studied beneficial bacteria in the gut and has a beneficial effect on hyperlipidemia, obesity, T2DM, and damage after myocardial infarction (MI). Studies have highlighted the potential of *L. rhamnosus* to reduce HFD-related metabolic disorders by cholesterol-lowering mechanism (176–179). Notably, *L. rhamnosus* GR-1 reduced the development of oxidative stress and chronic inflammation through the NF-κB signaling pathway and therefore reduced the formation of atherosclerotic plaque in ApoE-deficient mice fed with HFD (176). *L. rhamnosus* effectively regulated the abundance and diversity of intestinal flora in rats and zebrafish HFD models by increasing the abundance of Bacteriodetes. *L. rhamnosus* GG, a well-established probiotic strain, has a direct anti-obesity effect through the regulation of intestinal microbiota, particularly by decreasing the Firmicutes/Bacteroidetes ratio (180). In addition, *L. rhamnosus* GG can protect dyslipidemia and improve insulin sensitivity by inhibiting FXR and fibroblast growth factor 15 signaling and upregulating hepatic Cyp7A1 (79, 177, 181–183). *L. rhamnosus* GG supplementation was also found to be associated with stable HbA1c levels in healthy individuals (43). The cardioprotective effect of *L. rhamnosus* GG against high-fat high-fructose diet-induced obesity was associated with up-regulation of Nrf2-mediated antioxidant pathways (184). This strain also mitigated the development of obstructive sleep apnea-induced hypertension caused by a high-salt diet by regulating TMAO level and CD4^+^ T cell induced-type I inflammation (49). Compared with *L. rhamnosus* GG, *L. rhamnosus* NCDC17 can improve oral glucose tolerance and biochemical parameters; oxidative stress (TBARS); and CAT, SOD, and GSH-Px activities in the blood and liver and decrease the proportion of propionic acid in the cecum (185). Tissue weight assessment and atrial natriuretic peptide gene expression in MI rats given *L. rhamnosus* showed a substantial attenuation of left ventricular hypertrophy (LVH), and various ultrasound indicators reflected the improvement of left ventricular function (186). A clinical study also proved the effectiveness and safety of *Lactobacillus* supplementation in preventing cardiac remodeling after MI (42). Supplementation of *L. rhamnosus* in patients with coronary artery disease had beneficial effects on depression, anxiety, and inflammatory biomarkers (44, 45). In addition to the bacteria itself, the p75 protein isolated from *L. rhamnosus* GG (187) considerably reduced infarcts in rat cardiac tissues in a dose-dependent manner (188). Thus, the protein produced by lactobacilli also had a direct cardioprotective effect on ischemic damage and was no longer restricted to improving cardiovascular-related diseases through dietary intake. *L. rhamnosus* seems to be suitable for various people, we should dig out more strains like *L. rhamnosus* GG through clinical research.

### Other Lactobacilli

Eating habits are related to human health. For example, a high-salt diet can cause hypertension and other cardiovascular-related diseases. The intestinal flora perspective proved that a high-salt diet reduced the abundance of *Lactobacillus* in the gut, especially the consumption of *L. murinus*. Treatment with *L. murinus* prevented the salt-induced aggravation of actively induced experimental autoimmune encephalomyelitis and salt-sensitive hypertension in mice by modulating TH17 cells. In line with these findings, a moderate high-salt challenge in a pilot study in humans reduced the intestinal survival of *Lactobacillus* spp. and increased TH17 cells and blood pressure (21). High-salt intake is linked to the intestinal immune axis, and the intestinal microflora is an important condition against potential therapeutic targets for salt sensitivity that helps reduce the incidence of hypertension and reduces the risk of cardiovascular-related diseases. *In vitro* studies showed the cholesterol-lowering effect of strains with BSH activity, including *L. alimentarius, L. paraplantarum*, and *L. pentosus* (189–191). *Lactobacillus* supplementation with *L. buchneri, L. johnsonii*, and *L. mucosae* decreased serum cholesterol levels to prevent hypercholesterolemia (77, 192–195). Specifically, *L. johnsonii* BS15 markedly enhanced the population of Bacteroidetes and *Lactobacillus* spp. Moreover, the probiotic reduced the population of Enterobacteriaceae and the Firmicutes/Bacteroidetes ratio (192). The anti-obesity effect has been observed in *L. amylovorus, L. sakei*, and *L. salivarius*. The supplementation of *L. amylovorus* CP1563 had improved the anthropometric measurements and markers related to lipid and glucose metabolism and reduced the body fat of overweight and mildly obese individuals (24). Continuous ingestion of the fragmented CP1563 containing 10-hydroxyoctadecanoic acid also modulated the GM in pre-obese healthy subjects (25). *L. amylovorus* LKU4 exerted an anti-obesity effect on mice through facilitating browning of white adipocytes and increasing lactate levels (196). *L. sakei* CJLS03 treatment caused weight loss in people with obesity (46). *L. sakei* OK67 ameliorated HFD-induced blood glucose intolerance and obesity in mice by reducing inflammation and increasing the expression of colon tight junction proteins in mice (12, 197). Various obesity-associated biomarkers in the GM were also beneficially influenced by *L. sakei* administration (198, 199). *L. salivarius* Ls-33 modified fecal microbiota by increasing the *Bacteroides–Prevotella–Porphyromonas* group/Firmicutes ratio in obese adolescents (47). Viable probiotics (*Lactobacillus* and *Bifidobacterium*) had a cardioprotective effect on infarct-like myocardial injury by suppressing TNF-α and oxidative stress damage in a rat model; this effect had not been reported in the single treatment of *L. casei, L. bulgaricus*, and *L. acidophilus* (200). The co-supplementation of viable probiotics may be used as a new option for patients at risk of heart disease in the future. Despite evidence on the beneficial effects of *Lactobacillus* on the cardiovascular system has emerged, the impact of the other 17 *Lactobacillus* species on the management of cardiovascular-related diseases has not been elucidated.

## Candidate *Lactobacillus* Species for Relieving the Symptoms of Cardiovascular-Related Diseases

From the perspective of disease prevention, *L. casei, L. plantarum, L. fermentum, L. rhamnosus, L. reuteri*, and *L. paracasei* can treat the most cardiovascular-related diseases, and their corresponding mechanisms are the most extensive among the *Lactobacillus* species. This review provides guidance for the refinement of lactobacilli to treat several diseases effectively. Most lactobacilli prevent hyperlipidemia, hypercholesterolemia, and atherosclerosis through cholesterol-lowering mechanism via changes in BAs and TMAO. *L. acidophilus, L. amylovorus, L. brevis, L. casei, L. fermentum, L. paracasei, L. plantarum, L. reuteri, L. rhamnosus*, and *L. sakei* are examples of special bacteria for obesity that regulate lipid metabolism; inflammation; glucose metabolism; WAT energy metabolism; signaling pathways, including NF-κB and PPAR-α; and metabolites, including BAs and LPS. *L. casei, L. fermentum, L. paracasei, L. plantarum, L. reuteri, L. rhamnosus*, and *L. sakei* are special bacteria for T2DM and hyperglycemia that regulate glucose metabolism; inflammation; insulin sensitivity; pathways, including GPR43 and PI3K/Akt; and metabolites, including LPS, BAs, and SCFAs. *L. brevis, L. casei, L. fermentum, L. helveticus, L. murinus, L. paracasei, L. plantarum*, and *L. rhamnosus* are special bacteria for hypertension that regulate NO levels, ACEI activity, and LPS production.

As a primary and important factor in maintaining GM balance, the anti-pathogenic activity of lactobacilli has received a lot of attention. The massive numbers of bacteria with whole-genome sequence data have made possible the identification of an informative set of putative metabolite genes/gene clusters that encode antimicrobials across the genomes (Supplementary Figure 1). *L. paracasei, L. sakei, L. plantarum, L. casei, L. gasseri, L. alimentarius, L. coryniformis L. panis, L. crispatus, L. johnsonii, L. amylovorus, L. curvatus, L. rhamnosus*, and *L. helveticus* have been identified by genome mining as the most capable species for antimicrobial peptide production (Supplementary Table 2). Lactobacilli have the ability to produce different kinds of exopolysaccharides (EPSs) with a wide diversity of structures. EPS biosynthesis genes have been identified in most species within the *Lactobacillus* genus, such as *L. fermentum, L. reuteri, L. sakei*, and *L. plantarum* (201, 202). These species also have the potential to prevent and treat cardiovascular-related diseases by decreasing serum cholesterol, reducing the inflammatory response, and modulating GM composition (203–205). The predicted therapeutic candidates for cardiovascular-related diseases extend the existing reports of the *Lactobacillus* genus and are ready for experimental verification. Although numerous bacteria that lower cardiovascular risk are found in the literature, most bacteria have not been sequenced. Thus, genome mining cannot be performed. The possible disease treatment mechanism, target, and related products, which provide additional possibilities for the current research, can be speculated.

## Challenges and Future Directions of *Lactobacillus* Species in Therapeutic Research

### The Effectiveness and Safety of the Strains Is Still a Critical Issue

A proper evaluation of the products is essential before bringing *Lactobacillus* into routine usage. Animal and human studies have attempted to correct intestinal disorders by using probiotics, such as *L. rhamnosus* (180, 206) and *L. reuteri* (207), which were administered to animals under clinical and obesity factors, to target intestinal flora. Animal studies are consistent but occasionally fail to verify the results of human clinical studies. Animal experiments showed that *L. gasseri* BNR17 supplementation reduced body weight and white fat weight (208). By contrast, clinical trials showed that supplementation with *L. gasseri* BNR17 did not remarkably reduce body weight and waist and hip circumferences (209). *L. paracasei* had been used in animal experiments to reduce body weight and fat accumulation but did not affect metabolism in clinical studies (210). This discrepancy in outcome maybe because the current work is still in its early stages. Thus, apart from simple correlations, additional meaningful conclusions can be drawn from the data and lead to the transformation of animal experiments into human experiments. Animal experiment results that are consistent with clinical ones are reported. Moreover, most of the above studies are performed in strictly controlled animal models, which limits their potential applications in human subjects. Researchers must also need to consider lifestyle, age, genetic factors, different dietary conditions, and changes in the environment that affect the microbial composition. Therefore, clinical research is crucial. Some strains, such as *L. acidophilus* La5, *L. amylovorus* CP1563, *L. casei* Shirota, 01, *L. fermentum* ME-3, *L. gasseri* BNR17, SBT2055, *L. helveticus* LBK-16H, CM4, *L. plantarum* Lp299v, ECGC 13110402, OLL2712, Dad-13, *L. reuteri* NCIMB30242, DSM17938, ADR-1, ADR-3, V3401, *L. rhamnosus* GG, *L. sakei* CJLS03, and *L. salivarius* Ls-33 have been proven by clinical trials to be functional in improving cardiovascular-related diseases (Table 1). The scope of the topics covering legal edible bacteria can promote the study of clinical bacteria therapy.

### Mining Into the Bacteria to Explore Their Mechanism

Studies have shown that certain strains in a clearly balanced state are related to cardiovascular-related diseases. However, possible microbial communities that may be causally related to these diseases remain unknown. Various kinds of bacteria exist in the intestinal flora, and any intervention may lead to flora fluctuation. Therefore, finding the target bacteria is difficult, and the technology of knocking out some bacteria *in vivo* research must be developed (59, 211). However, gene-editing technology invalidates the hypothetical target metabolites to verify its effect. Researchers can also attempt to separate the metabolites directly for animal clinical trials with the development of the isolation and purification of bacterial metabolites. The identification of the normal metabolic pathway given in this review or screening the target protein or pathway by high-throughput screening technologies, such as microarray, transcriptomic, and metabonomic technology, and then verifying the results by laboratory experiments for further mechanism research will be popular.

### Therapeutic Potential of Natural Products by Targeting Lactobacilli

Polysaccharides, saponins, and flavonoids, as the main active components in daily food, are difficult to be directly digested and absorbed by the human body. They play remarkable pharmacological roles depending on the transformation by intestinal microorganisms. Lactobacilli produce various substances, such as β-glucosidase and β-galactosidase, making them useful as fermentation tools and influence the function and activity of natural products. The metabolic pathway of ginsenoside bioconversion using enzymes and microbial fermentation has been reviewed, and *L. rhamnosus* GG, *L. delbrueckii, L. acidophilus, L. plantarum*, and *L. brevis* play a major role (212). For instance, compound K (C-K; 20-O-D-glucopyranosyl-20(S)-protopanaxadiol) is a novel ginsenoside metabolite, with anti-inflammatory, anti-atherosclerosis, and anti-diabetic activity, formed by intestinal lactobacilli β-glucosidase and does not occur naturally in ginseng (213). *L. rhamnosus* C6 strain showed higher β-glucosidase activity as well as biotransformation of isoflavones from glycones (daidzin and genistin) to aglycones (daidzein and genistein), which indicated an inverse relationship between the incidence of cardiovascular diseases (214). A series of novel phenolic galactosides with high antioxidant capacity was achieved by β-galactosidase from *L. bulgaricus* L3 (215). Lactobacilli participate in producing SCFAs by the fermentation of polysaccharide, which is generated during the glycolytic pathway (216). Thus, the activity of lactobacilli in the intestines has a significant effect on the digestion of food and medicine, and prevents the occurrence and development of cardiovascular-related diseases.

## Conclusions

The World Health Organization reports that 30% of deaths worldwide are caused by cardiovascular-related diseases and predicts that these diseases will remain the leading cause of death in the next 20 years. These diseases will introduce considerable physical and economic burden on humans (https://www.who.int/health-topics/cardiovascular-diseases); thus, additional attention has been provided to cardiovascular-related diseases. Studies have shown that the development of cardiovascular-related diseases is closely related to the structure and function of GM (4). The concept that probiotics could improve cardiovascular-related diseases is emerging. In recent years, changes in intestinal microbial community, metabolites, and their link to cardiovascular-related diseases have made GM a potential new target for treatment. Therapeutic approaches with *Lactobacillus* could directly maintain GM homeostasis and regulate functional metabolites, including TMAO, SCFAs, BAs, and LPS, which further help to reduce the risks of high lipid cholesterol, immune inflammation, and oxidative stress.

The category of beneficial *Lactobacillus* strains referred to in this review provides experimental data for clinical use. Lactobacilli have promising metabolites; thus, a wide range of *Lactobacillus* candidates for future research on the improvement of cardiovascular-related diseases have been figured out. In addition to current therapeutic interventions, including fecal microbial transplantation, dietary interventions, and prebiotic and antibiotic interventions (81, 217–219), *Lactobacillus* therapy still represents an exciting frontier in the prevention and treatment of cardiovascular-related diseases.

## Author Contributions

XZha and XZho collected the literature's data and wrote the manuscript. XL helped draw mechanism's figures. XW and XG supervised and revised the manuscript. All authors have read and agreed to the published version of the manuscript.

## Conflict of Interest

The authors declare that the research was conducted in the absence of any commercial or financial relationships that could be construed as a potential conflict of interest.

## Abbreviations

ACEI: Angiotensin-converting enzyme inhibitors
AM: Alveolar macrophages
Apo A-I: Apolipoprotein A-I
Apo B: Apolipoprotein B
BAs: Bile acids
BSH: Bile salt hydrolase
CHD: Coronary heart disease
Cpt1a: Carnitine palmitoyl transferase 1a
Cyp7A1: Cytochrome P450 a1
DC: Diabetic cardiomyopathy
DCA: Deoxycholic acid
DOCA: Deoxycorticosterone
EFSA: European Food Safety Authority
FGF: Fibroblast growth factor
FXR: Farnesoid X receptor
GM: Gut microbiota
GRAS: Generally recognized as safe
GSH: Glutathione
HbA1c: Glycated hemoglobin
HDL-C: High density lipoprotein cholesterol
HF: Heart failure
HFD: High-fat diet
HMG-CoA: 3-hydroxy-3-methyl glutaryl coenzyme A
ICAM: Intercellular adhesion molecule
Insig: Insulin induced gene
LDL: Low density lipoprotein
LDL-C: Low density lipoprotein cholesterol
LDL-R: Low density lipoprotein receptor
LPS: Lipopolysaccharide
LVH: Left ventricular hypertrophy
LXR: Liver-X-receptors
MCP-1: Monocyte chemotactic protein-1
MI: Myocardial infarction
NE: Norepinephrine
NO: Nitric oxide
NPC1L1: Niemann-Pick C1-like 1
QPS: Qualified presumption of safety
SCFAs: Short chain fatty acids
SOD: Superoxide dismutase
TAS: Total antioxidant status
TBARS: Thiobarbituric acid reactive substances
TC: Total cholesterol
T2DM: Type 2 diabetes
TG: Triacylglycerols
TLC: Therapeutic lifestyle changes
TMAO: Trimethylamine N-oxide
TSST-1: Toxic shock syndrome toxin-1
VaD: Vascular dementia
VCAM: Vascular cell adhesion molecule
WAT: White adipose tissue
WHO: World Health Organization.
