# Supplementary material for: Therapeutic and Improving Function of Lactobacilli in the Prevention and Treatment of Cardiovascular-Related Diseases: A Novel Perspective From Gut Microbiota
Source: Front Nutr. 2021 Jun 7;8:693412. doi: 10.3389/fnut.2021.693412 (PMC8215129; doi:10.3389/fnut.2021.693412)
Supplement: Supplementary file 1 [file Data_Sheet_1.pdf]

Supplementary Material

1 Supplementary Figures and Tables

1.1 Supplementary Figures

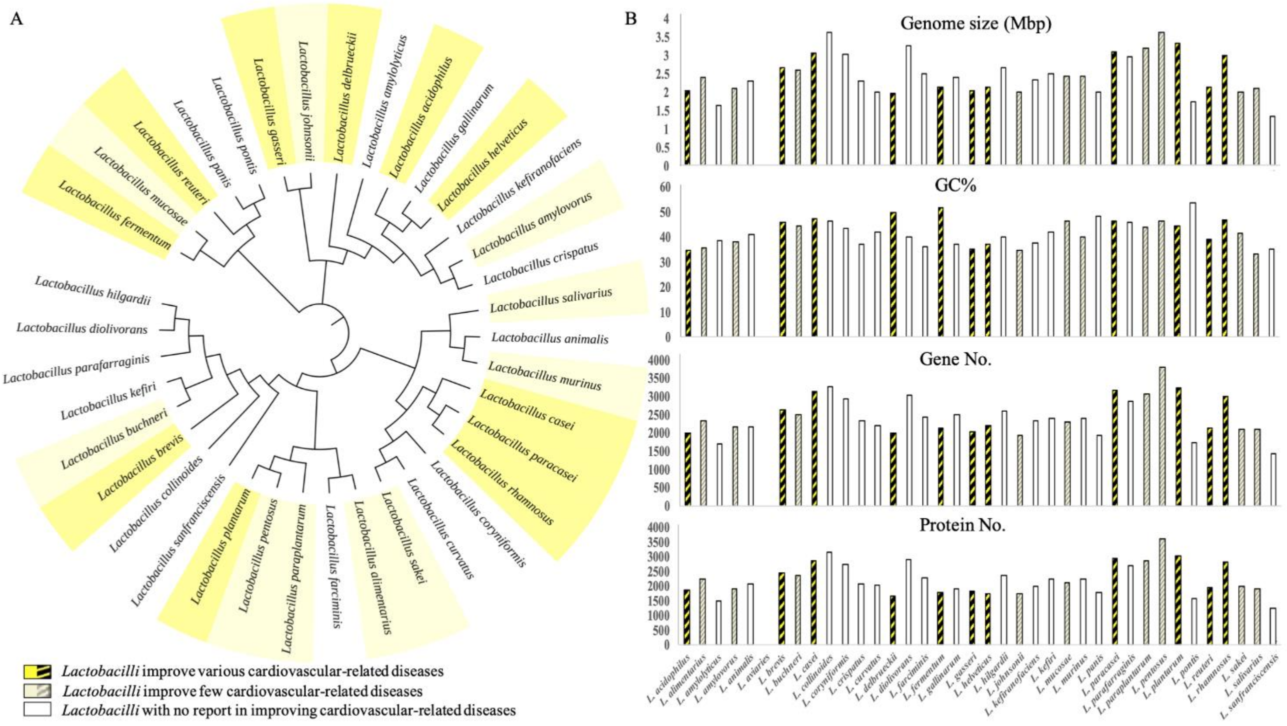

Supplementary Figure 1. Genome overview of various lactobacilli. (A) phylogenetic tree of *Lactobacillus* species studied in this review. The phylogenetic tree was constructed using MEGA 6.0 software and modified by iTOL based on the reference genome of each species. (B) genome information including genome size, GC%, gene and protein numbers.

1.2 Supplementary Tables

Table S1 List of lactobacilli in this review with safety certification

No.	*Lactobacillus* species	Safety certification (• presents getting permit)		
		CDFA	U.S. FDA	EFSA
1	*L. acidophilus*	•	•	•
2	*L. alimentarius*			•
3	*L. amylolyticus*			•
4	*L. amylovorus*			•
5	*L. animalis*			•
6	*L. aviaries*			•
7	*L. brevis*			•
8	*L. buchneri*			•
9	*L. casei*	•	•	•
10	*L. collinoides*			•
11	*L. coryniformis*			•
12	*L. crispatus*	•		•
13	*L. curvatus*		•	•
14	*L. delbrueckii*	•		•
15	*L. diolivorans*			•
16	*L. farciminis*			•
17	*L. fermentum (cellobiosus)*	•	•	•

18	*L. gallinarum*			•
19	*L. gasseri*	•		•
20	*L. helveticus*	•	•	•
21	*L. hilgardii*			•
22	*L. johnsonii*	•		•
23	*L. kefiranofaciens*			•
24	*L. kefiri*			•
25	*L. mucosae*			•
26	*L. murinus*			
27	*L. panis*			•
28	*L. paracasei*	•	•	•
29	*L. parafarraginis*			•
30	*L. paraplantarum*			•
31	*L. pentosus*			•
32	*L. plantarum*	•	•	•
33	*L. pontis*			•
34	*L. reuteri*	•	•	•
35	*L. rhamnosus*	•	•	•
36	*L. sakei*			•
37	*L. salivarius*	•		•
38	*L. sanfranciscensis*			•

Table S2 Known and putative antimicrobial gene clusters identified in 404 lactobacilli genomes (reported in Genbank)

No.	*Lactobacillus* (*No.* of strains)	Bacteriocin (*No.* of BGCs)				Other putative antimicrobials (*No.* of BGCs)			Total *No.* (*No.* of BGCs)
		Putative Class I	Known Class II and III	Other Putative	Total	PKs	NRPs	Other	
1	*L. acidophilus (7)*	Lanthipeptide (2)		1 (5)	2 (7)				2 (7)
2	*L. alimentarius (1)*								
3	*L. amylolyticus (2)*								
4	*L. amylovorus (5)*	Lanthipeptide (1)	Gassericin T (3); Linocin M18 (1)		3 (5)				3 (5)
5	*L. animalis (1)*	Thiopeptide (1)		1 (3)	2 (4)	T3PKS (1)			3 (5)
6	*L. aviaries (0)*								
7	*L. brevis (22)*	Lanthipeptide (7)	Linocin M18 (11)		2 (18)	T3PKS (22)			3 (40)

8	*L. buchneri (3)*		Linocin M18 (2)	1 (1)	2 (3)	T3PKS (3)		3 (6)
9	*L. casei (6)*		Lactococcin 972 (1);<br>Holin-like (1)	3 (11)	5 (13)			5 (13)
10	*L. collinoides (0)*							
11	*L. coryniformis (3)*							
12	*L. crispatus (4)*	LAP (1)	Gassericin T (3);	2 (4)	4 (8)			4 (8)
13	*L. curvatus (13)*		ThmA (1);<br>Lactobin A/cerein 7B (1);<br>Enterocin A (1)	1 (1)	4 (4)			4 (4)
14	*L. delbrueckii (24)*	Lanthipeptide (16)	Lactococcin 972 (3)		2 (19)		NRPS -like (4)	3 (23)

15	*L. diolivorans (0)*								
16	*L. farciminis (3)*								
17	*L. fermentum (25)*							Betalactone (1)	1 (1)
18	*L. gallinarum (1)*								
19	*L. gasseri (4)*		Gassericin T (2);<br>Gassericin S (1)	3 (4)	5 (7)				5 (7)
20	*L. helveticus (18)*	LAP (4)		1 (2)	2 (6)		NRPS (4)	Arylpolyene (1)	4 (11)
21	*L. hilgardii (1)*					T3PKS (1)			1 (1)
22	*L. johnsonii (11)*	Lanthipeptide (4)	Gassericin T (7);		2 (11)				2 (11)
23	*L. kefiranofaciens (2)*	Lanthipeptide (1)			1 (1)			Arylpolyene (2)	2 (3)
24	*L. kefiri (1)*					T3PKS (1)			1 (1)

25	*L. mucosae (1)*								
26	*L. murinus (3)*			2 (8)	2 (8)	T3PKS (2)			3 (10)
27	*L. panis (0)*								
28	*L. paracasei (32)*		Holin-like (6)	2 (42)	3 (48)				3 (48)
29	*L. parafarraginis (0)*								
30	*L. paraplantarum (3)*			1 (3)	1 (3)	T3PKS (3)		Phytoene (3)	3 (9)
31	*L. pentosus (6)*	RaS-RiPP (4)			1 (4)	T3PKS (6)			2 (10)
32	*L. plantarum (122)*			1 (119)	1 (119)	T3PKS (113)	NpsA (2)	Phytoene (119)	4 (353)
33	*L. pontis (1)*					T3PKS (1)			1 (1)
34	*L. reuteri (16)*			1 (4)	1 (4)	T3PKS (16)			2 (20)
35	*L. rhamnosus (27)*		Lactobin/cerein 7B (13)	2 (8)	3 (21)	T3PKS (19)			4 (40)

36	*L. sakei (25)*		Sakacin-P (2);	1 (1)	2 (3)	Calicheamicin (11);<br>T3PKS (1)			4 (15)
37	*L. salivarius (9)*			1 (1)	1 (1)	T3PKS (9)			2 (10)
38	*L. sanfranciscensis (2)*					T3PKS (2)		Phytoene (1)	2 (3)
	Total (404)	4 (41)	9 (59)	24 (217)	37 (317)	2 (211)	3 (10)	3 (127)	45 (665)

A detailed prediction of the gene clusters of bacteriocins, NRPs, PKs and other antimicrobials is provided by anti-SMASH (http://antismash.secondarymetabolites.org), a web server and stand-alone tool for the automatic genomic identification and analysis of biosynthetic gene clusters (BGCs).
