# Supplementary figures and images for: Therapeutic and Improving Function of Lactobacilli in the Prevention and Treatment of Cardiovascular-Related Diseases: A Novel Perspective From Gut Microbiota
Source: Front Nutr. 2021 Jun 7;8:693412. doi: 10.3389/fnut.2021.693412 (PMC8215129; doi:10.3389/fnut.2021.693412)

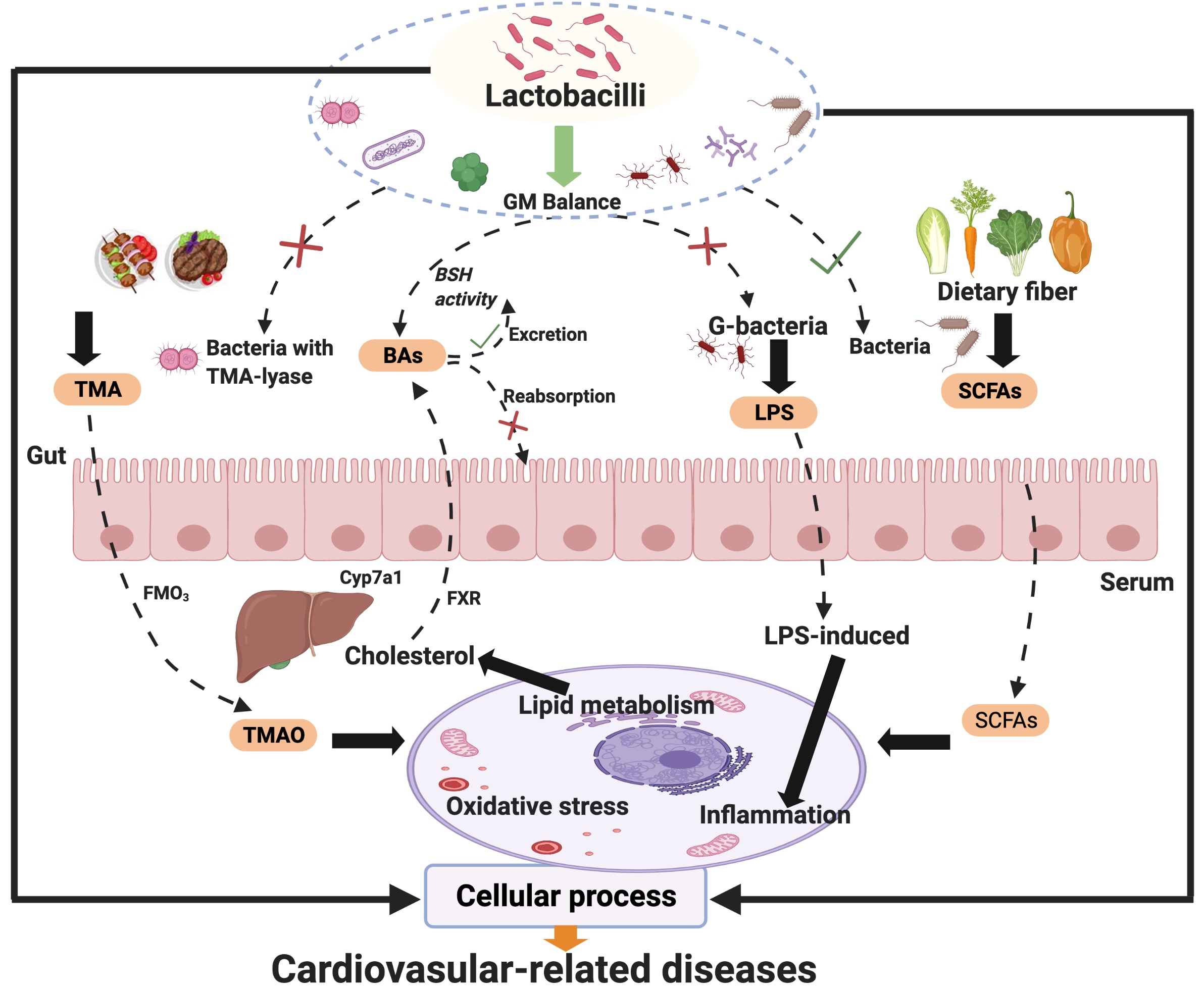

Supplement: Supplementary file 2 [file Image_1.TIF]
